# The genome sequence of the common hop, *Humulus lupulus* L.

**DOI:** 10.12688/wellcomeopenres.24025.1

**Published:** 2025-04-23

**Authors:** Maarten J. M. Christenhusz, Lawrence Percival-Alwyn, Klara Hajdu, John Connell, Ilia J. Leitch, Royal Botanic Gardens Kew Genome Acquisition Lab, Darwin Tree of Life Barcoding collective, Plant Genome Sizing collective, Wellcome Sanger Institute Tree of Life Management, Samples and Laboratory team, Wellcome Sanger Institute Scientific Operations: Sequencing Operations, Wellcome Sanger Institute Tree of Life Core Informatics team, Tree of Life Core Informatics collective, Darwin Tree of Life Consortium

**Affiliations:** 1 Royal Botanic Gardens Kew, Richmond, England, UK; 2 Curtin University, Perth, Western Australia, Australia; 3 National Institute of Agricultural Botany, Cambridge, England, UK; 4 Wye Hops, Canterbury, England, UK

**Keywords:** Humulus lupulus, Common hop, Hops, genome sequence, chromosomal, Rosales

## Abstract

We present a genome assembly from a female *Humulus lupulus* plant (Common hop, Hops; Streptophyta; Magnoliopsida; Rosales; Cannabaceae). The genome sequence has a total length of 2,488.10 megabases. Most of the assembly (99.39%) is scaffolded into 10 chromosomal pseudomolecules, including the X sex chromosome. The mitochondrial and plastid genome assemblies have lengths of 400.02 kilobases and 153.77 kilobases, respectively. Gene annotation of this assembly by Ensembl identified 32,487 protein-coding genes.

## Species taxonomy

Eukaryota; Viridiplantae; Streptophyta; Streptophytina; Embryophyta; Tracheophyta; Euphyllophyta; Spermatophyta; Magnoliopsida; Mesangiospermae; eudicotyledons; Gunneridae; Pentapetalae; rosids; fabids; Rosales; Cannabaceae; *Humulus*; *Humulus lupulus* L. (NCBI:txid3486)

## Background

The common or European hop ( *Humulus lupulus* L. var. *lupulus*) is a herbaceous, dioecious climbing plant in the Cannabaceae family, characterised by perennial roots and annual stems that die back each winter. The genus *Humulus* is currently recognised as comprising at least seven species ( POWO, 2025), though earlier classifications often listed three ( *H. lupulus*, *H. scandens* and *H. yunnanensis*). Of these, only *H. lupulus* produces the resinous cones used in brewing. Some species are restricted to North America.

The evolutionary origin of *Humulus* remains uncertain. While earlier classifications suggested an East Asian origin, recent phylogenomic analyses indicate a possible Central Asian origin, given its sister relationship to *Cannabis* ( Fu *et al.*, 2023). However, phylogenetic studies of Cannabaceae remain limited by sparse sampling.

Native to temperate regions across North America and Eurasia, hops are cultivated globally, ideally between latitudes 35° and 55° in both hemispheres, where climatic conditions favour growth cycles ( Small, 2016). In Britain, hops predominantly grow in southern and eastern counties, notably Kent, due to suitable soils and climate. Wild hops are found along rivers, woodlands, and disturbed areas.

Hop plants sprout from underground rhizomes each spring, growing up to 10 m before dying back in winter. They are bines, not vines, meaning they climb by twining around a support rather than using tendrils or suckers. They climb clockwise using tiny hairs on their stems to grip onto vertical structures for support which, for cultivation, traditionally consists of poles and wirework. As short-day photoperiod plants, hops initiate flowering when day lengths shorten below a critical threshold. Dormancy is induced by shortening photoperiods in autumn, with regrowth triggered by increased temperatures and longer photoperiods in spring ( Bauerle, 2019).

Male and female hop plants are morphologically distinct: females produce cone-like inflorescences called strobili, which contain lupulin glands rich in essential oils and resins; males form loose panicles of flowers that release wind-borne pollen ( Padgitt-Cobb *et al.*, 2023). Only the female plants are used in commercial hop production and are propagated clonally via shoots or rhizomes to maintain desired genotypes ( Neve, 1991).

The phytochemicals synthesised in the lupulin glands, including prenylated flavonoids (e.g., xanthohumol), bitter acids (humulone and lupulone), essential oils, and terpenoids, are critical for flavouring and bittering beer ( Takoi *et al.*, 2010). These compounds confer the characteristic aroma and bitterness that have been integral to beer production for centuries.

As a high-value crop, hop cultivation is primarily driven by the brewing industry, although hop metabolites are increasingly recognised for medicinal potential due to their sedative, estrogenic, antioxidant, anti-inflammatory, and antimicrobial properties ( Girisa *et al.*, 2021). The global demand for hops has risen with the popularity of craft breweries, with the market size expected to reach USD 14.35 billion by 2032 ( Global Market Insights, 2023).

Despite their “Least Concern” status on the IUCN Red List, hops face pressures from climate change and diseases such as downy mildew ( *Pseudoperonospora humuli*), powdery mildew ( *Podosphaera macularis* ssp. *humuli*), and Verticillium wilt ( *Verticillium nonalfalfae*) ( Hajdu, 2022). Additionally, hop damson aphid infestations ( *Phorodon humuli*), which vector viral diseases like hop mosaic virus and hop latent viroid, pose significant threats. Wild hop populations are also threatened by hybridisation with exotic cultivars in fields and gardens ( Hampton *et al.*, 2001).

*Humulus lupulus* is diploid (2 *n* = 20 with XX/XY sex chromosomes) and exhibits complex genetics that can hinder genomic characterisation. Unusual transmission genetics arise from chromosome rearrangements, aneuploidy, and complex multivalent structures formed during meiosis, leading to non-Mendelian segregation patterns ( Easterling *et al.*, 2018). Different karyotype groups with distinct chromosomal rearrangements exist within hop germplasm, causing breeding difficulties due to nonviable progeny from incompatible crosses.

In this study, we present a high-quality chromosome-level genome sequence of a wild-sourced female *Humulus lupulus* from Surrey, England, generated as part of the Darwin Tree of Life Project ( Blaxter *et al.*, 2022). This resource, together with the previously-published chromosome-level genome assembly for the Cascade cultivar of *H. lupulus* ( Padgitt-Cobb *et al.,* 2023), will enhance our ability to identify and distinguish between haplotypes within populations and supports genotyping methods like skim sequencing. It will also facilitate association mapping studies to uncover genes underlying important agronomic traits such as disease resistance, yield, and quality. As more chromosome-level assemblies become available, understanding synteny and large-scale genomic rearrangements like inversions and translocations becomes feasible. This genomic resource paves the way for constructing a hop pan-genome, further aiding breeders in developing cultivars resilient to pathogens and climate change.

## Genome sequence report

### Sequencing data

**Figure 1.**
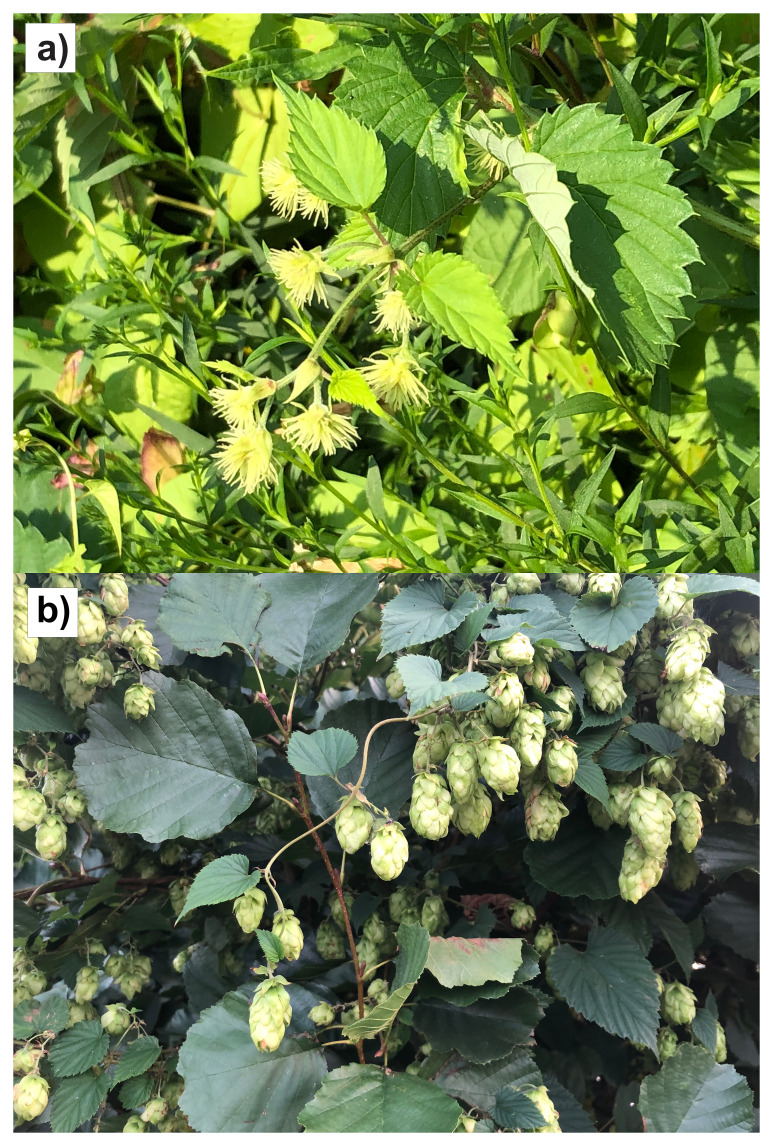
Photographs of the female *Humulus lupulus* (drHumLupu1, KDTOL10038) specimen from which samples were taken for genome sequencing: **a**) in flower **b**) in fruit later in the year.

The genome of a specimen of *Humulus lupulus* ( Figure 1) was sequenced using Pacific Biosciences single-molecule HiFi long reads, generating 83.23 Gb (gigabases) from 7.09 million reads. GenomeScope analysis of the PacBio HiFi data estimated the haploid genome size at 2,520.75 Mb, with a heterozygosity of 0.33% and repeat content of 54.95%. These values provide an initial assessment of genome complexity and the challenges anticipated during assembly. Based on this estimated genome size, the sequencing data provided approximately 32.0x coverage of the genome. Using flow cytometry, the genome size (1C-value) of the sampled *Humulus lupulus* was estimated to be 2.93 pg, equivalent to 2,870 Mb. Chromosome conformation Hi-C sequencing produced 565.94 Gb from 3,747.96 million reads.

Table 1 summarises the specimen and sequencing information, including the BioProject, study name, BioSample numbers, and sequencing data for each technology.

**Table 1.** Specimen and sequencing data for *Humulus lupulus*.

Project information			
**Study title**	Humulus lupulus (European hop)		
**Umbrella BioProject**	PRJEB64076		
**Species**	*Humulus lupulus*		
**BioSpecimen**	SAMEA7522047		
**NCBI taxonomy ID**	3486		
Specimen information			
**Technology**	**ToLID**	**BioSample accession**	**Organism part**
**PacBio long read sequencing**	drHumLupu1	SAMEA7522103	leaf
**Hi-C sequencing**	drHumLupu1	SAMEA7522102	leaf
**RNA sequencing**	drHumLupu1	SAMEA7522103	leaf
Sequencing information			
**Platform**	**Run accession**	**Read count**	**Base count (Gb)**
**Hi-C Illumina NovaSeq 6000**	ERR11679392	3.75e+09	565.94
**PacBio Sequel IIe**	ERR11673235	4.17e+05	6.73
**PacBio Revio**	ERR11809134	6.67e+06	76.49
**RNA Illumina NovaSeq X**	ERR12861039	6.50e+07	9.82

### Assembly statistics

The primary haplotype was assembled, and contigs corresponding to an alternate haplotype were also deposited in INSDC databases. The assembly was improved by manual curation, which corrected 37 misjoins or missing joins. These interventions reduced the total assembly length by 1.17%, decreased the scaffold count by 4.46%, and decreased the scaffold N50 by 4.68%. The final assembly has a total length of 2,488.10 Mb in 319 scaffolds, with 129 gaps, and a scaffold N50 of 251.53 Mb ( Table 2).

**Table 2.** Genome assembly data for *Humulus lupulus*.

Genome assembly		
Assembly name	drHumLupu1.1	
Assembly accession	GCA_963169125.1	
*Alternate haplotype accession*	*GCA_963169115.1*	
Assembly level for primary assembly	chromosome	
Span (Mb)	2,488.10	
Number of contigs	448	
Number of scaffolds	319	
Longest scaffold (Mb)	312.51	
Assembly metric	Measure	*Benchmark*
Contig N50 length	29.24 Mb	*≥ 1 Mb*
Scaffold N50 length	251.53 Mb	*= chromosome N50*
Consensus quality (QV)	Primary: 64.8; alternate: 64.3; combined: 64.5	*≥ 40*
*k*-mer completeness	Primary: 90.83%; alternate: 82.13%; combined: 98.68%	*≥ 95%*
BUSCO *	C:98.1%[S:95.0%,D:3.1%], F:0.3%,M:1.6%,n:2,326	*S > 90%; D < 5%*
Percentage of assembly mapped to chromosomes	99.39%	*≥ 90%*
Sex chromosomes	X	*localised homologous pairs*
Organelles	Mitochondrial genome: 400.02 kb, Plastid genome: 153.77 kb	*complete single alleles*

* BUSCO scores based on the eudicots_odb10 BUSCO set using version 5.5.0. C = complete [S = single copy, D = duplicated], F = fragmented, M = missing, n = number of orthologues in comparison.

**Figure 2.**
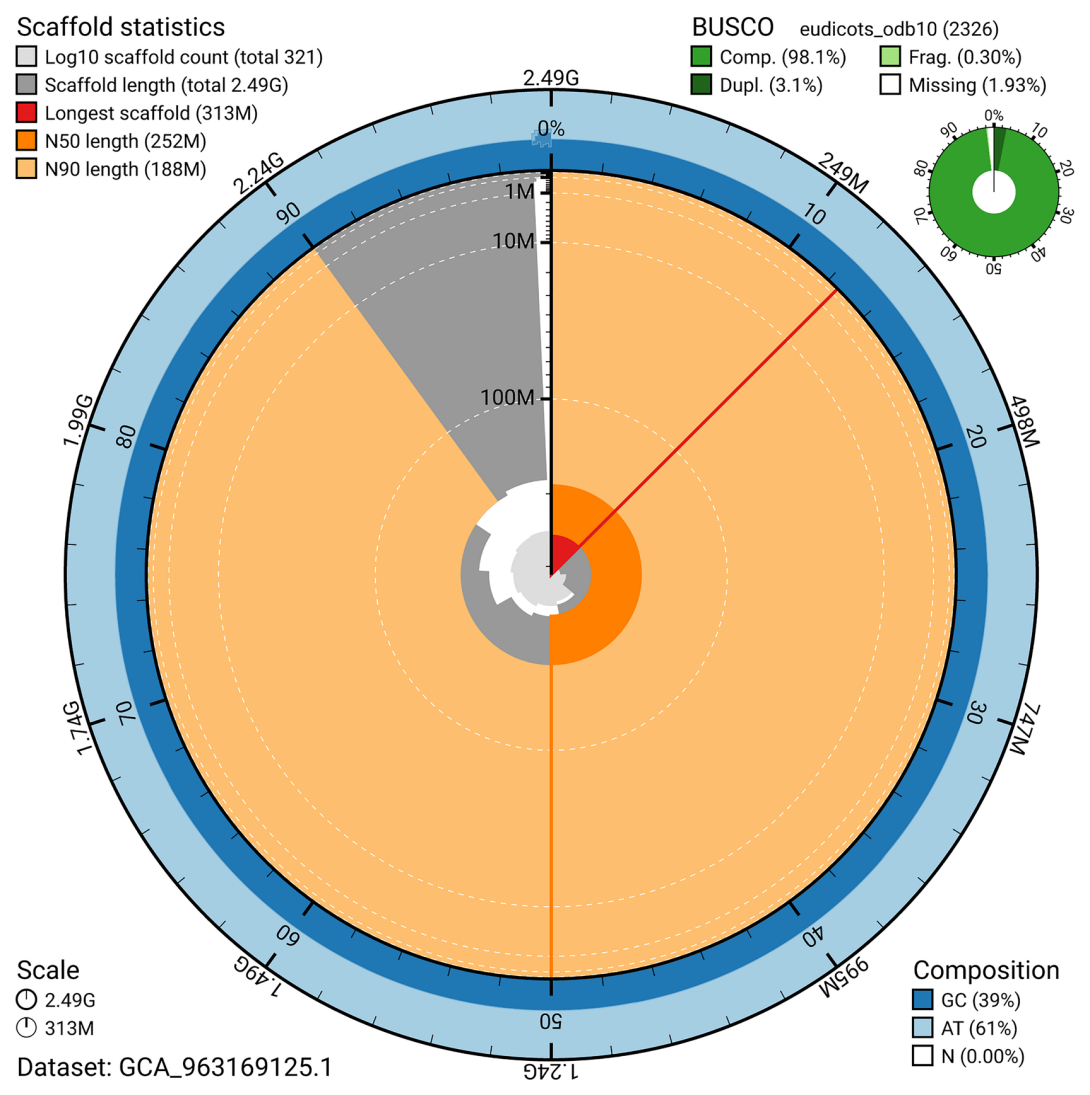
Genome assembly of *Humulus lupulus*, drHumLupu1.1: metrics. The BlobToolKit snail plot provides an overview of assembly metrics and BUSCO gene completeness. The circumference represents the length of the whole genome sequence, and the main plot is divided into 1,000 bins around the circumference. The outermost blue tracks display the distribution of GC, AT, and N percentages across the bins. Scaffolds are arranged clockwise from longest to shortest and are depicted in dark grey. The longest scaffold is indicated by the red arc, and the deeper orange and pale orange arcs represent the N50 and N90 lengths. A light grey spiral at the centre shows the cumulative scaffold count on a logarithmic scale. A summary of complete, fragmented, duplicated, and missing BUSCO genes in the eudicots_odb10 set is presented at the top right. An interactive version of this figure is available at https://blobtoolkit.genomehubs.org/view/GCA_963169125.1/dataset/GCA_963169125.1/snail.

**Figure 3.**
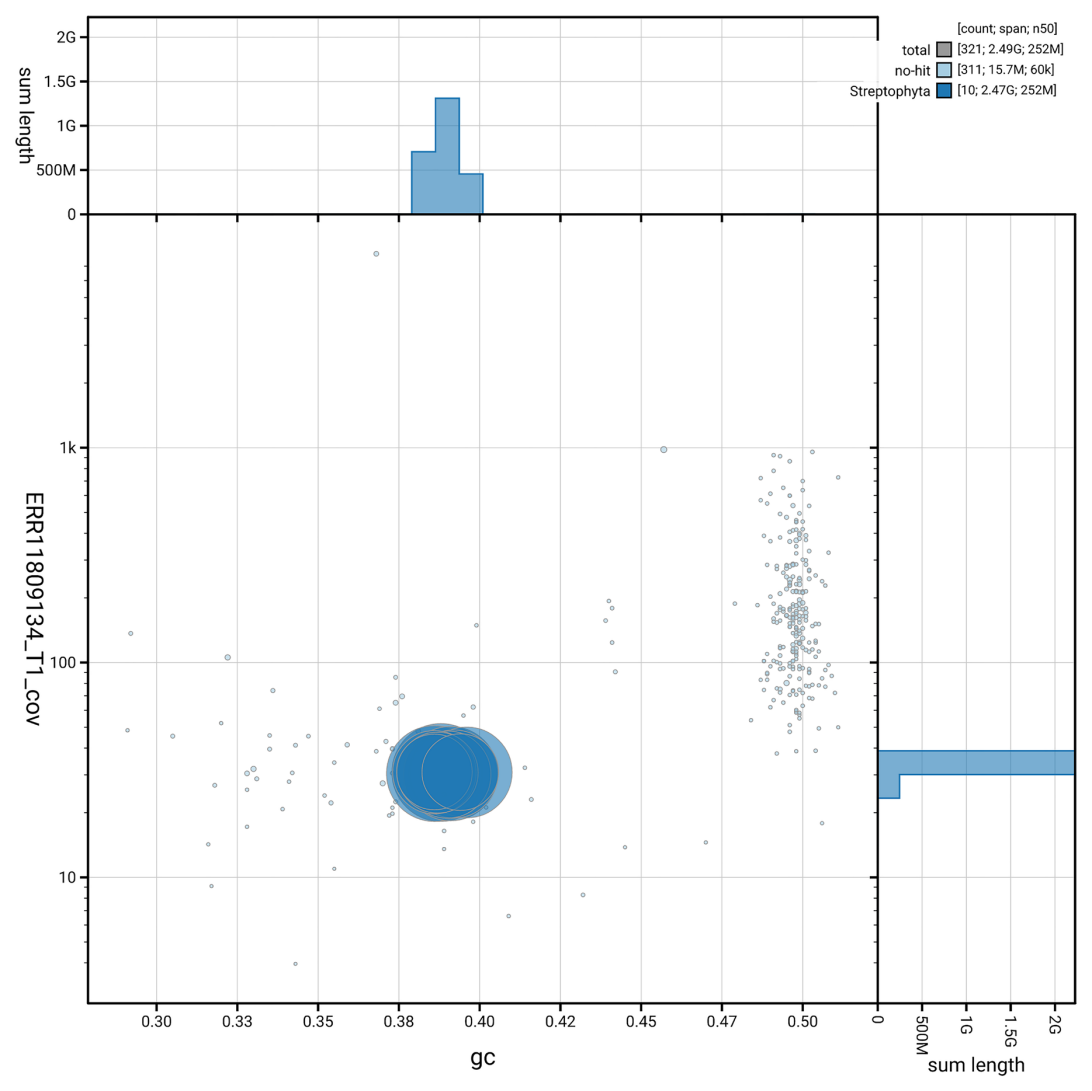
Genome assembly of *Humulus lupulus*, drHumLupu1.1: BlobToolKit GC-coverage plot. Blob plot showing sequence coverage (vertical axis) and GC content (horizontal axis). The circles represent scaffolds, with the size proportional to scaffold length and the colour representing phylum membership. The histograms along the axes display the total length of sequences distributed across different levels of coverage and GC content. An interactive version of this figure is available at https://blobtoolkit.genomehubs.org/view/GCA_963169125.1/dataset/GCA_963169125.1/blob.

**Figure 4.**
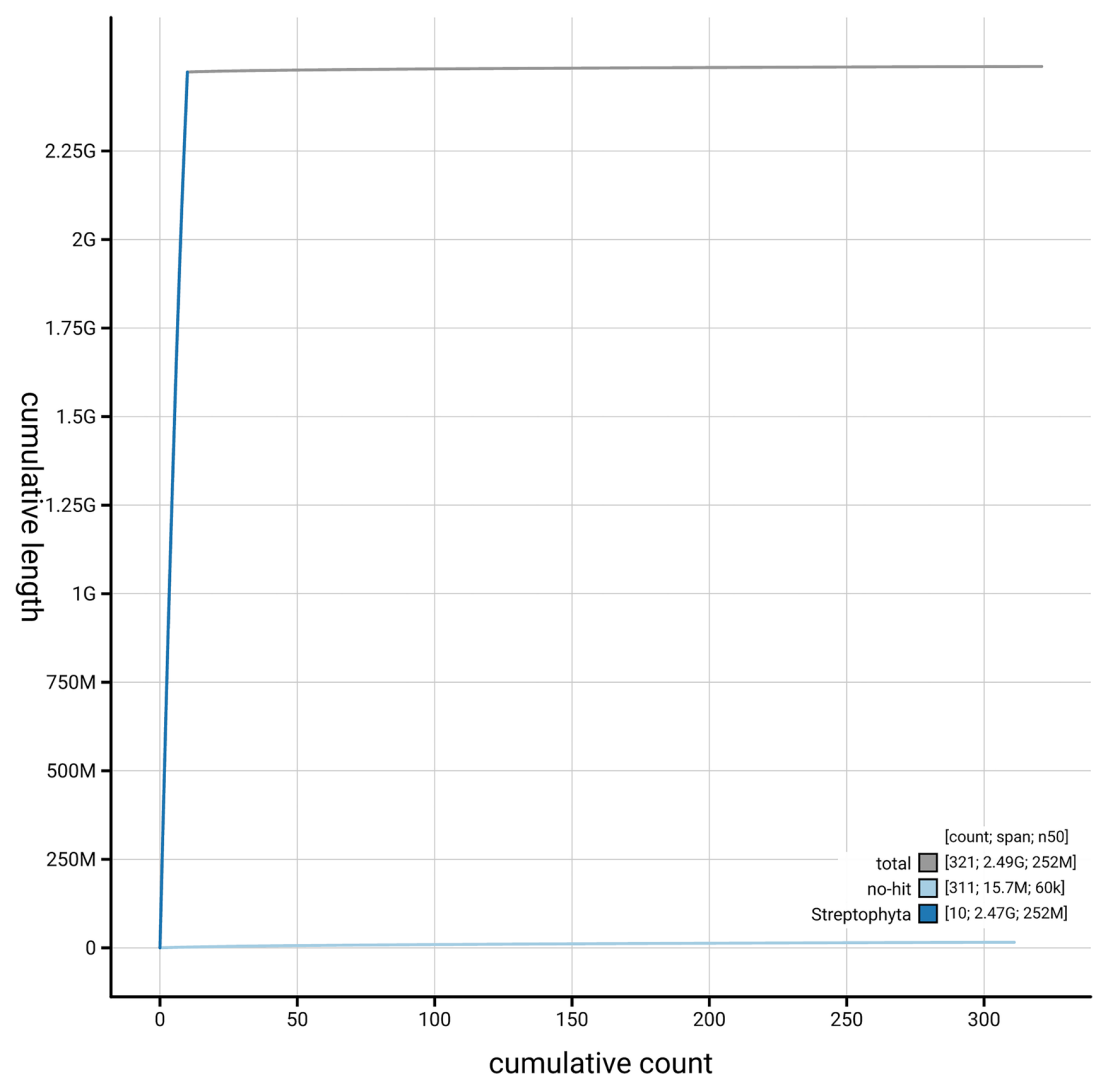
Genome assembly of *Humulus lupulus,* drHumLupu1.1: BlobToolKit cumulative sequence plot. The grey line shows cumulative length for all scaffolds. Coloured lines show cumulative lengths of scaffolds assigned to each phylum using the buscogenes taxrule. An interactive version of this figure is available at https://blobtoolkit.genomehubs.org/view/GCA_963169125.1/dataset/GCA_963169125.1/cumulative.

The snail plot in Figure 2 provides a summary of the assembly statistics, indicating the distribution of scaffold lengths and other assembly metrics. Figure 3 shows the distribution of scaffolds by GC proportion and coverage. Figure 4 presents a cumulative assembly plot, with separate curves representing different scaffold subsets assigned to various phyla, illustrating the completeness of the assembly.

**Figure 5.**
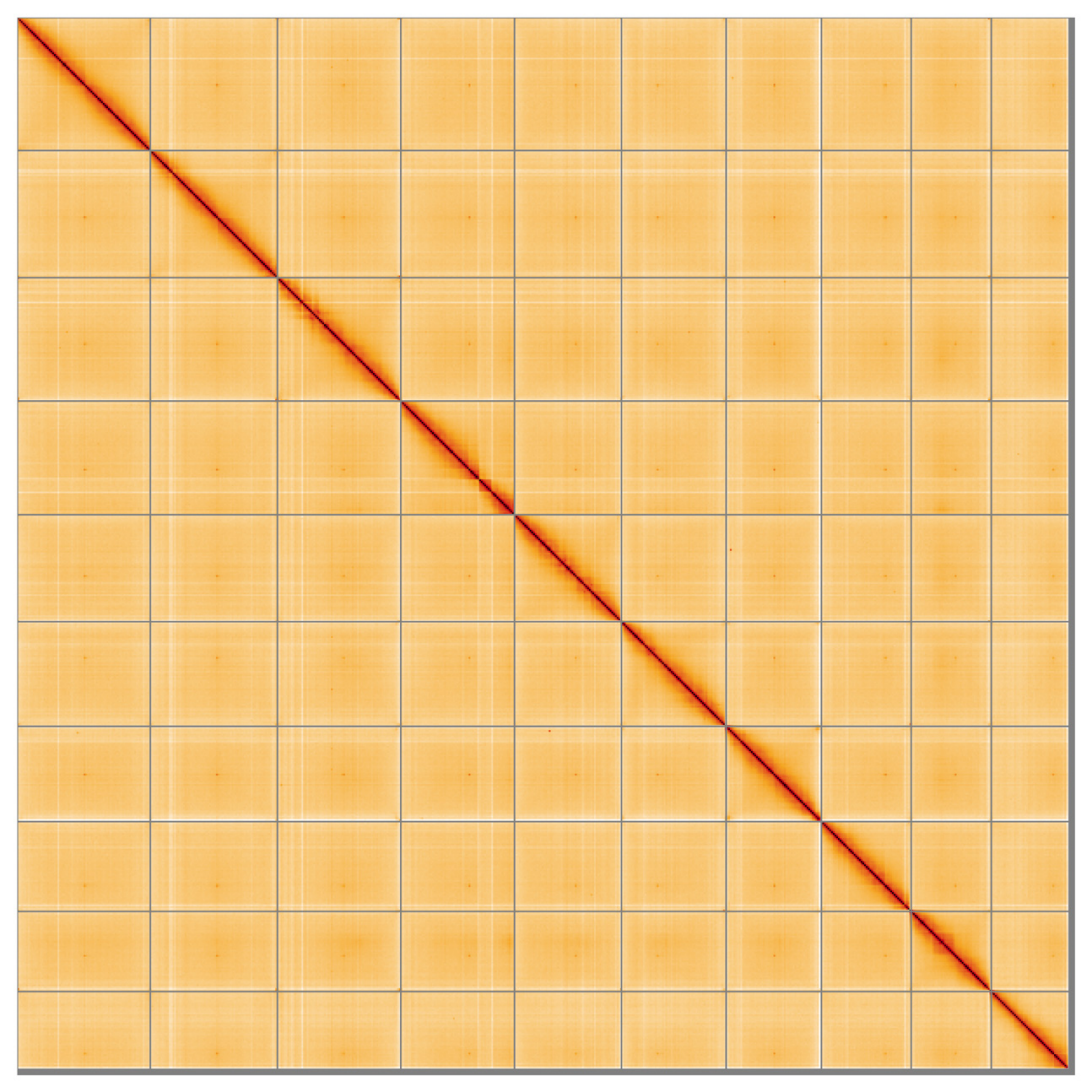
Genome assembly of *Humulus lupulus,* drHumLupu1.1: Hi-C contact map of the drHumLupu1.1 assembly, visualised using HiGlass. Chromosomes are shown in order of size from left to right and top to bottom. An interactive version of this figure may be viewed at https://genome-note-higlass.tol.sanger.ac.uk/l/?d=A4aJuAB0TC6RoMS6BQyDZw.

Most of the assembly sequence (99.39%) was assigned to 10 chromosomal-level scaffolds, representing 9 autosomes and the X sex chromosome. These chromosome-level scaffolds, confirmed by Hi-C data, are named according to size ( Figure 5; Table 3). During curation, the X chromosome was identified by alignment with the hopbase Cascade genome ( Padgitt-Cobb *et al.*, 2023).

**Table 3.** Chromosomal pseudomolecules in the genome assembly of *Humulus lupulus*, drHumLupu1.

INSDC accession	Name	Length (Mb)	GC%
OY720647.1	1	312.51	39
OY720648.1	2	298.99	38.5
OY720649.1	3	289.95	39
OY720651.1	4	251.53	39
OY720652.1	5	246.04	39
OY720653.1	6	224.07	38.5
OY720654.1	7	210.93	38.5
OY720655.1	8	183.3	38.5
OY720656.1	9	188.39	39.5
OY720650.1	X	267.21	39.5
OY720657.1	MT	0.4	45.5

The mitochondrial and plastid genomes were also assembled. These sequences are included as contigs in the multifasta file of the genome submission and as standalone records in GenBank.

### Assembly quality metrics

The estimated Quality Value (QV) and *k*-mer completeness metrics, along with BUSCO completeness scores, were calculated for each haplotype and the combined assembly. The QV reflects the base-level accuracy of the assembly, while *k*-mer completeness indicates the proportion of expected *k*-mers identified in the assembly. BUSCO scores provide a measure of completeness based on benchmarking universal single-copy orthologues.

The primary haplotype has a QV of 64.8, and the combined primary and alternate assemblies achieve an estimated QV of 64.5. The *k*-mer recovery for the primary haplotype is 90.83%, and for the alternate haplotype 82.13%; the combined primary and alternate assemblies have a *k*-mer recovery of 98.68%. BUSCO analysis using the eudicots_odb10 reference set ( *n* = 2,326) identified 98.1% of the expected gene set (single = 95.0%, duplicated = 3.1%).

Table 2 provides assembly metric benchmarks adapted from Rhie *et al.* (2021) and the Earth BioGenome Project (EBP) Report on Assembly Standards September 2024. The assembly achieves the EBP reference standard of **7.C.Q64**.

## Genome annotation report

The *Humulus lupulus* genome assembly (GCA_963169125.1) was annotated externally by Ensembl at the European Bioinformatics Institute (EBI). This annotation includes 77,693 transcribed mRNAs from 32,487 protein-coding and 27,085 non-coding genes. The average transcript length is 2,870.69. There are 1.30 coding transcripts per gene and 4.00 exons per transcript. For further information about the annotation, please refer to https://rapid.ensembl.org/Humulus_lupulus_GCA_963169125.1/Info/Index.

## Methods

### Sample acquisition, DNA barcoding and genome size estimation

Metadata collection for samples adhered to the Darwin Tree of Life project standards described by Lawniczak *et al*. (2022). A *Humulus lupulus* (specimen ID KDTOL10038, ToLID drHumLupu1) was collected from Canbury Gardens, Kingston Upon Thames, Surrey, United Kingdom (latitude 51.42, longitude –0.31) on 2020-08-12. The specimen was collected and identified by Maarten J. M. Christenhusz (Royal Botanic Gardens Kew; RBG Kew; collection number MC9035) and preserved by freezing at –80 °C. The herbarium voucher associated with the sequenced plant is K001400618 and is deposited in the herbarium of RBG Kew (K).

The initial species identification was verified by an additional DNA barcoding process according to the framework developed by Twyford *et al*. (2024). Part of the plant specimen was preserved in silica gel desiccant ( Chase & Hills, 1991). A DNA extraction from the dried plant was amplified by PCR for standard barcode markers, with the amplicons sequenced and compared to public sequence databases including GenBank and the Barcode of Life Database (BOLD). The barcode sequences for this specimen are openly available on BOLD ( Ratnasingham & Hebert, 2007). Following whole genome sequence generation, DNA barcodes were also used alongside the initial barcoding data for sample tracking through the genome production pipeline at the Wellcome Sanger Institute (WSI; Twyford *et al.*, 2024). The standard operating procedures for the Darwin Tree of Life barcoding have been deposited on protocols.io ( Beasley *et al.*, 2023).

The genome size was estimated by flow cytometry using the fluorochrome propidium iodide and following the ‘one-step’ method as outlined in Pellicer *et al.* (2021). For this species, the General Purpose Buffer (GPB) supplemented with 3% PVP and 0.08% (v/v) beta-mercaptoethanol was used for isolation of nuclei ( Loureiro *et al.*, 2007). The internal calibration standard was Pisum sativum *‘*Ctirad’ with an assumed 1C-value of 4,445 Mb ( Doležel *et al.*, 1998).

### Nucleic acid extraction

The workflow for high molecular weight (HMW) DNA extraction at the WSI Tree of Life Core Laboratory includes sample preparation and homogenisation, DNA extraction, fragmentation and purification. Detailed protocols are available on protocols.io ( Denton *et al.*, 2023). In sample preparation, the drHumLupu1 sample was weighed and dissected on dry ice ( Jay *et al.*, 2023). Leaf tissue was homogenised by cryogenic bead beating ( Jackson & Howard, 2023). HMW DNA was extracted using the Automated Plant MagAttract v2 protocol ( Todorovic *et al.*, 2023). HMW DNA was sheared into an average fragment size of 12–20 kb in a Megaruptor 3 system ( Bates *et al.*, 2023). Sheared DNA was purified by solid-phase reversible immobilisation, using AMPure PB beads to eliminate shorter fragments and concentrate the DNA ( Strickland *et al.*, 2023). The concentration of the sheared and purified DNA was assessed using a Nanodrop spectrophotometer and Qubit Fluorometer and Qubit dsDNA High Sensitivity Assay kit. Fragment size distribution was evaluated by running the sample on the FemtoPulse system.

RNA was extracted from leaf tissue of drHumLupu1 in the Tree of Life Laboratory at the WSI using the RNA Extraction: Automated MagMax™ *mir*Vana protocol ( do Amaral *et al.*, 2023). The RNA concentration was assessed using a Nanodrop spectrophotometer and a Qubit Fluorometer using the Qubit RNA Broad-Range Assay kit. Analysis of the integrity of the RNA was done using the Agilent RNA 6000 Pico Kit and Eukaryotic Total RNA assay.

### Hi-C sample preparation

Hi-C data were generated from the leaf tissue using the Arima-HiC v2 kit at the WSI Scientific Operations core. Tissue was finely ground using cryoPREP and then subjected to nuclei isolation. Nuclei were isolated using a modified protocol of the Qiagen QProteome Cell Compartment Kit where only CE1 and CE2 buffers are used in combination with QiaShredder spin columns. After isolation, the nuclei were fixed using 37% formaldehyde solution to crosslink the DNA. The crosslinked DNA was then digested using the restriction enzyme master mix. The 5’-overhangs were then filled in and labelled with biotinylated nucleotides and proximally ligated. An overnight incubation was carried out for enzymes to digest remaining proteins and for crosslinks to reverse. A clean up was performed with SPRIselect beads prior to library preparation. DNA concentration was quantified using the Qubit Fluorometer v4.0 and Qubit HS Assay Kit according to the manufacturer’s instructions.

### Library preparation and sequencing

Library preparation and sequencing were performed at the WSI Scientific Operations core.

***PacBio HiFi***

At a minimum, samples were required to have an average fragment size exceeding 8 kb and a total mass over 400 ng to proceed to the low input SMRTbell Prep Kit 3.0 protocol (Pacific Biosciences, California, USA). Libraries were prepared using the SMRTbell Prep Kit 3.0 (Pacific Biosciences, California, USA) as per the manufacturer’s instructions. The kit includes the reagents required for end repair/A-tailing, adapter ligation, post-ligation SMRTbell bead cleanup, and nuclease treatment. Following the manufacturer’s instructions, size selection and clean up was carried out using diluted AMPure PB beads (Pacific Biosciences, California, USA). DNA concentration was quantified using the Qubit Fluorometer v4.0 (Thermo Fisher Scientific) with Qubit 1X dsDNA HS assay kit and the final library fragment size analysis was carried out using the Agilent Femto Pulse Automated Pulsed Field CE Instrument (Agilent Technologies) and gDNA 55kb BAC analysis kit.

Samples were sequenced on a Revio instrument (Pacific Biosciences, California, USA). Prepared libraries were normalised to 2 nM, and 15 μL was used for making complexes. Primers were annealed and polymerases were hybridised to create circularised complexes, according to manufacturer’s instructions. The complexes were purified with the 1.2X clean up with SMRTbell beads. The purified complexes were then diluted to the Revio loading concentration, in the range 200–300 pM, and spiked with a Revio sequencing internal control. Samples were sequenced on Revio 25M SMRT cells. The SMRT link software, a PacBio web-based end-to-end workflow manager, was used to set-up and monitor the run, as well as perform primary and secondary analysis of the data upon completion.

***Hi-C***

For Hi-C library preparation, DNA was fragmented to a size of 400 to 600 bp using a Covaris E220 sonicator. The DNA was then enriched, barcoded, and amplified using the NEBNext Ultra II DNA Library Prep Kit (New England Biolabs) following manufacturer’s instructions. Hi-C sequencing was performed using paired-end sequencing with a read length of 150 bp on an Illumina NovaSeq 6000 instrument.

***RNA***

Poly(A) RNA-Seq libraries were constructed using the NEB Ultra II RNA Library Prep kit, following the manufacturer’s instructions. RNA sequencing was performed on the Illumina NovaSeq X instrument.

### Genome assembly, curation and evaluation

***Assembly***

Prior to assembly of the PacBio HiFi reads, a database of *k*-mer counts ( *k* = 31) was generated from the filtered reads using FastK. GenomeScope2 ( Ranallo-Benavidez *et al.*, 2020) was used to analyse the *k*-mer frequency distributions, providing estimates of genome size, heterozygosity, and repeat content.

The HiFi reads were assembled using Hifiasm in Hi-C phasing mode ( Cheng *et al.*, 2021; Cheng *et al.*, 2022), resulting in a pair of haplotype-resolved assemblies. The Hi-C reads were mapped to the primary contigs using bwa-mem2 ( Vasimuddin *et al.*, 2019). The contigs were further scaffolded using the provided Hi-C data ( Rao *et al.*, 2014) in YaHS ( Zhou *et al.*, 2023) using the --break option for handling potential misassemblies. The scaffolded assemblies were evaluated using Gfastats ( Formenti *et al.*, 2022), BUSCO ( Manni *et al.*, 2021) and MERQURY.FK ( Rhie *et al.*, 2020). The organelle genomes were assembled using OATK ( Zhou, 2023).

***Assembly curation***

The assembly was decontaminated using the Assembly Screen for Cobionts and Contaminants (ASCC) pipeline. Flat files and maps used in curation were generated via the TreeVal pipeline ( Pointon *et al.*, 2023). Manual curation was conducted primarily in PretextView ( Harry, 2022) and HiGlass ( Kerpedjiev *et al.*, 2018), with additional insights provided by JBrowse2 ( Diesh *et al.*, 2023). Scaffolds were visually inspected and corrected as described by Howe *et al*. (2021). Any identified contamination, missed joins, and mis-joins were amended, and duplicate sequences were tagged and removed. The curation process is documented at https://gitlab.com/wtsi-grit/rapid-curation.

***Evaluation of assembly quality***

The Merqury.FK tool ( Rhie *et al.*, 2020), run in a Singularity container ( Kurtzer *et al.*, 2017), was used to evaluate *k*-mer completeness and assembly quality for the primary and alternate haplotypes using the *k*-mer databases ( *k* = 31) computed prior to genome assembly. The analysis outputs included assembly QV scores and completeness statistics.

A Hi-C contact map was produced for the final version of the assembly. The Hi-C reads were aligned using bwa-mem2 ( Vasimuddin *et al.*, 2019) and the alignment files were combined using SAMtools ( Danecek *et al.*, 2021). The Hi-C alignments were converted into a contact map using BEDTools ( Quinlan & Hall, 2010) and the Cooler tool suite ( Abdennur & Mirny, 2020). The contact map was visualised in HiGlass ( Kerpedjiev *et al.*, 2018).

The blobtoolkit pipeline is a Nextflow port of the previous Snakemake Blobtoolkit pipeline ( Challis *et al.*, 2020). It aligns the PacBio reads in SAMtools and minimap2 ( Li, 2018) and generates coverage tracks for regions of fixed size. In parallel, it queries the GoaT database ( Challis *et al.*, 2023) to identify all matching BUSCO lineages to run BUSCO ( Manni *et al.*, 2021). For the three domain-level BUSCO lineages, the pipeline aligns the BUSCO genes to the UniProt Reference Proteomes database ( Bateman *et al.*, 2023) with DIAMOND ( Buchfink *et al.*, 2021) blastp. The genome is also split into chunks according to the density of the BUSCO genes from the closest taxonomic lineage, and each chunk is aligned to the UniProt Reference Proteomes database with DIAMOND blastx. Genome sequences with no hits are chunked with seqtk and aligned to the NT database with blastn ( Altschul *et al.*, 1990). The blobtools suite combines all these outputs into a blobdir for visualisation.

The blobtoolkit pipeline was developed using nf-core tooling ( Ewels *et al.*, 2020) and MultiQC ( Ewels *et al.*, 2016), relying on the Conda package manager, the Bioconda initiative ( Grüning *et al.*, 2018), the Biocontainers infrastructure ( da Veiga Leprevost *et al.*, 2017), as well as the Docker ( Merkel, 2014) and Singularity ( Kurtzer *et al.*, 2017) containerisation solutions.

Table 4 contains a list of relevant software tool versions and sources.

**Table 4.** Software tools: versions and sources.

Software tool	Version	Source
BEDTools	2.30.0	https://github.com/arq5x/bedtools2
BLAST	2.14.0	ftp://ftp.ncbi.nlm.nih.gov/blast/executables/blast+/
BlobToolKit	4.3.9	https://github.com/blobtoolkit/blobtoolkit
BUSCO	5.5.0	https://gitlab.com/ezlab/busco
bwa-mem2	2.2.1	https://github.com/bwa-mem2/bwa-mem2
Cooler	0.8.11	https://github.com/open2c/cooler
DIAMOND	2.1.8	https://github.com/bbuchfink/diamond
fasta_windows	0.2.4	https://github.com/tolkit/fasta_windows
FastK	427104ea91c78c3b8b8b49f1a7d6bbeaa869ba1c	https://github.com/thegenemyers/FASTK
Gfastats	1.3.6	https://github.com/vgl-hub/gfastats
GoaT CLI	0.2.5	https://github.com/genomehubs/goat-cli
Hifiasm	0.19.5-r587	https://github.com/chhylp123/hifiasm
HiGlass	44086069ee7d4d3f6f3f0012569789ec138f42b84aa44357826c0b6753eb28de	https://github.com/higlass/higlass
MerquryFK	d00d98157618f4e8d1a9190026b19b471055b22e	https://github.com/thegenemyers/MERQURY.FK
Minimap2	2.24-r1122	https://github.com/lh3/minimap2
MultiQC	1.14, 1.17, and 1.18	https://github.com/MultiQC/MultiQC
Nextflow	23.04.1	https://github.com/nextflow-io/nextflow
OATK	0.9	https://github.com/c-zhou/oatk
PretextView	0.2.5	https://github.com/sanger-tol/PretextView
samtools	1.19.2	https://github.com/samtools/samtools
sanger-tol/ascc	-	https://github.com/sanger-tol/ascc
sanger-tol/blobtoolkit	0.5.1	https://github.com/sanger-tol/blobtoolkit
Seqtk	1.3	https://github.com/lh3/seqtk
Singularity	3.9.0	https://github.com/sylabs/singularity
TreeVal	1.2.0	https://github.com/sanger-tol/treeval
YaHS	1.2a.2	https://github.com/c-zhou/yahs

### Wellcome Sanger Institute – Legal and Governance

The materials that have contributed to this genome note have been supplied by a Darwin Tree of Life Partner. The submission of materials by a Darwin Tree of Life Partner is subject to the **‘Darwin Tree of Life Project Sampling Code of Practice’**, which can be found in full on the Darwin Tree of Life website here. By agreeing with and signing up to the Sampling Code of Practice, the Darwin Tree of Life Partner agrees they will meet the legal and ethical requirements and standards set out within this document in respect of all samples acquired for, and supplied to, the Darwin Tree of Life Project.

Further, the Wellcome Sanger Institute employs a process whereby due diligence is carried out proportionate to the nature of the materials themselves, and the circumstances under which they have been/are to be collected and provided for use. The purpose of this is to address and mitigate any potential legal and/or ethical implications of receipt and use of the materials as part of the research project, and to ensure that in doing so we align with best practice wherever possible. The overarching areas of consideration are:

•   Ethical review of provenance and sourcing of the material

•   Legality of collection, transfer and use (national and international)

Each transfer of samples is further undertaken according to a Research Collaboration Agreement or Material Transfer Agreement entered into by the Darwin Tree of Life Partner, Genome Research Limited (operating as the Wellcome Sanger Institute), and in some circumstances other Darwin Tree of Life collaborators.

## Data Availability

European Nucleotide Archive: Humulus lupulus (European hop). Accession number PRJEB64076; https://identifiers.org/ena.embl/PRJEB64076. The genome sequence is released openly for reuse. The *Humulus lupulus* genome sequencing initiative is part of the Darwin Tree of Life (DToL) project. All raw sequence data and the assembly have been deposited in INSDC databases. Raw data and assembly accession identifiers are reported in Table 1.

## Author information

Members of the Royal Botanic Gardens Kew Genome Acquisition Lab are listed here: https://doi.org/10.5281/zenodo.12625079.

Members of the Plant Genome Sizing collective are listed here: https://doi.org/10.5281/zenodo.7994306.

Members of the Darwin Tree of Life Barcoding collective are listed here: https://doi.org/10.5281/zenodo.12158331

Members of the Wellcome Sanger Institute Tree of Life Management, Samples and Laboratory team are listed here: https://doi.org/10.5281/zenodo.12162482.

Members of Wellcome Sanger Institute Scientific Operations: Sequencing Operations are listed here: https://doi.org/10.5281/zenodo.12165051.

Members of the Wellcome Sanger Institute Tree of Life Core Informatics team are listed here: https://doi.org/10.5281/zenodo.12160324.

Members of the Tree of Life Core Informatics collective are listed here: https://doi.org/10.5281/zenodo.12205391.

Members of the Darwin Tree of Life Consortium are listed here: https://doi.org/10.5281/zenodo.4783558.
