# Supplementary material for: The genome sequence of the common hop, Humulus lupulus L
Source: Wellcome Open Res. 2025 Apr 23;10:210. [Version 1] doi: 10.12688/wellcomeopenres.24025.1 (PMC12171724; doi:10.12688/wellcomeopenres.24025.1)
Supplement: Peer review history

# Reviewer report: Reviewer response for version 1

**DOI:** 10.21956/wellcomeopenres.26507.r123754

**Published:** 2025-06-16

**Authors:** Jose Ramon Planta

**Affiliations:** 1 University of the Philippines Diliman, Quezon city, Philippines

Overall, this is a very good genome assembly.

 1. Please provide a brief rationale explaining why the combined assembly is included in the analysis, in addition to the primary and alternate assemblies. Also, a description in the Methods section on how the combined assembly was generated can be included. 

 2. I don't believe Figure 4 is very important. It could be replaced with a table that details annotations (e.g., number of tRNAs, rRNAs, etc.) for the organellar genomes.

Are sufficient details of methods and materials provided to allow replication by others?

Yes

Is the rationale for creating the dataset(s) clearly described?

Yes

Are the datasets clearly presented in a useable and accessible format?

Yes

Are the protocols appropriate and is the work technically sound?

Yes

Reviewer Expertise:

Maize genetics, grass genomics

I confirm that I have read this submission and believe that I have an appropriate level of expertise to confirm that it is of an acceptable scientific standard.

# Reviewer report: Reviewer response for version 1

**DOI:** 10.21956/wellcomeopenres.26507.r123004

**Published:** 2025-05-30

**Authors:** Jernej Jakse

**Affiliations:** 1 University of Ljubljana, Ljubljana, Ljubljana, Slovenia

The paper presents a whole-chromosome-level assembly of the common hop (Humulus lupulus), a valuable medicinal and industrial species. The data provided are of significant importance for evolutionary studies as well as for supporting research and breeding efforts in hop cultivation.

 Overall, the paper is well written, and all necessary data typically expected in genome assembly reports are included and supported by appropriate tables and figures.

 However, I noted a few discrepancies:

 -According to the Methods section, RNA sequencing was performed, but no results or analyses related to that dataset are presented.

 -Figure 4 appears hard to understand.

 -The plastid genome is not included on the NCBI genome assembly page (https://www.ncbi.nlm.nih.gov/datasets/genome/GCF_963169125.1/), although it is available as a separate record.

 -A direct link to the BioProject PRJEB64075 (https://www.ncbi.nlm.nih.gov/bioproject/PRJEB64075), where the raw data are deposited, would be helpful for readers.

 Congratulations to the authors on providing a high-quality chromosome-scale assembly of the hop genome.

Are sufficient details of methods and materials provided to allow replication by others?

Yes

Is the rationale for creating the dataset(s) clearly described?

Yes

Are the datasets clearly presented in a useable and accessible format?

Yes

Are the protocols appropriate and is the work technically sound?

Yes

Reviewer Expertise:

plant genetics, bioinformatics, plant pathogen interactions

I confirm that I have read this submission and believe that I have an appropriate level of expertise to confirm that it is of an acceptable scientific standard.
